# Anaemia among females in child-bearing age: Relative contributions, effects and interactions of α- and β-thalassaemia

**DOI:** 10.1371/journal.pone.0206928

**Published:** 2018-11-02

**Authors:** Sachith Mettananda, Marius Suranjan, Veani Fernando, Tiran Dias, Chamila Mettananda, Rexan Rodrigo, Lakshman Perera, Richard Gibbons, Anuja Premawardhena, Douglas Higgs

**Affiliations:** 1 Department of Paediatrics, Faculty of Medicine, University of Kelaniya, Ragama, Sri Lanka; 2 Colombo North Teaching Hospital, Ragama, Sri Lanka; 3 Department of Obstetrics and Gynaecology, Faculty of Medicine, University of Kelaniya, Ragama, Sri Lanka; 4 Department of Pharmacology, Faculty of Medicine, University of Kelaniya, Ragama, Sri Lanka; 5 Department of Medicine, Faculty of Medicine, University of Kelaniya, Ragama, Sri Lanka; 6 MRC Molecular Haematology Unit, MRC Weatherall Institute Molecular Medicine, University of Oxford, Oxford, United Kingdom; CSIR-Foood Research Institute, GHANA

## Abstract

**Introduction:**

Anaemia in women during pregnancy and child bearing age is one of the most common global health problems. Reasons are numerous, but in many cases only minimal attempts are made to elucidate the underlying causes. In this study we aim to identify aetiology of anaemia in women of child bearing age and to determine the relative contributions, effects and interactions of α- and β-thalassaemia in a region of the world where thalassaemia is endemic.

**Methods:**

A cross sectional study was conducted at the Colombo North Teaching Hospital of Sri Lanka. The patient database of deliveries between January 2015 and September 2016 at University Obstetrics Unit was screened to identify women with anaemia during pregnancy and 253 anaemic females were randomly re-called for the study. Data were collected using an interviewer-administered questionnaire and haematological investigations were done to identify aetiologies.

**Results:**

Out of the 253 females who were anaemic during pregnancy and were re-called, 8 were excluded due to being currently pregnant. Of the remaining 245 females, 117(47.8%) remained anaemic and another 22(9.0%) had non-anaemic microcytosis. Of anaemic females, 28(24.8%) were iron deficient, 40(35.4%) had low-normal serum ferritin without fulfilling the criteria for iron deficiency,18(15.3%) had β-haemoglobinopathy trait and 20(17.0%) had α-thalassaemia trait. Of females who had non-anaemic microcytosis, 14(66.0%) had α-thalassaemia trait. In 4 females, both α- and β-thalassaemia trait coexist. These females had higher levels of haemoglobin (p = 0.06), MCV (p<0.05) and MCH (p<0.01) compared to individuals with only β-thalassaemia trait. A significantly higher proportion of premature births (p<0.01) and lower mean birth weights (p<0.05) were observed in patients with α-thalassaemia trait.

**Conclusions:**

Nearly one third of anaemic females in child bearing age had thalassaemia trait of which α-thalassemia contributes to a majority. Both α- and β-thalassaemia trait can co-exist and have ameliorating effects on red cell indices in heterozygous states. α-Thalassaemia trait was significantly associated with premature births and low birth weight. It is of paramount importance to investigate the causes of anaemia in women of child bearing age and during pregnancy in addition to providing universal iron supplementation.

## Introduction

Anaemia is one of the most common public health problems in the world. It is estimated that over 1.9 billion people of the world’s population suffer from anaemia across the globe[1]. The highest prevalences were reported in south Asia and central, west, and east sub-Saharan African regions [2]. The prevalence is also high in certain vulnerable age groups including children under 5 years of age, pregnant women and women of child bearing age[3].

The global prevalence of anaemia in pregnancy is estimated to be 41% which varies from 5.7% in the USA to 75% in the Gambia[4]. Anaemia in pregnancy is associated with a number of obstetric complications which lead to increased risk of maternal morbidity and mortality[4]. Similarly, anaemia during pregnancy, especially when it is severe, is associated with adverse perinatal outcomes that include increased risk of foetal distress, preterm birth and small for gestational age[5]. Additionally, babies born to anaemic mothers are known to develop iron deficiency and anaemia during late infancy.

Causes of anaemia during pregnancy are diverse however, in many instances the aetiology is not determined. Most health authorities including the World Health Organisation attribute anaemia in pregnant women in low- and middle income countries solely to iron deficiency. Therefore, iron supplementation is given to all pregnant women without attempting to find a cause. However, in regions where there is a high prevalence of haemoglobinopathies, α- and β-thalassaemia could contribute to a large proportion of anaemia in pregnant women and women of child bearing age[2, 6]. This is of particular importance due to the fact that many individuals with haemoglobinopathies are iron replete hence, do not require iron supplementation and indeed may be at risk of iron overload[7]. In some occasions, inappropriate supplementations could lead to deleterious effects associated with increased iron availability leading to an increased risk of infection[8] and adverse effects on the gut microbiome[9]. Therefore, in this study we aimed to identify the aetiology of anaemia in women of child bearing age and to determine the relative contribution of α- and β-thalassaemia in a region of the world where thalassaemia is endemic.

## Methods

### Participant recruitment

A cross sectional study was conducted at the Colombo North Teaching Hospital (CNTH), Ragama, Sri Lanka from June to December 2017. CNTH is one of the eight University Hospitals and is the main tertiary referral centre for parts of the Western and North Western Provinces of the country. The University obstetrics unit of the CNTH maintains an electronic data base of all deliveries (North Colombo Obstetric Database [NORCOD]) and births taking place at the unit. We reviewed data of haemoglobin levels at the first antenatal clinic visit of all pregnant women whose babies were delivered at the unit between January 2015 to September 2016 to identify mothers who were anaemic during pregnancy. Anaemia in pregnancy was defined as haemoglobin level <11.5 g/dl. All females who were anaemic during pregnancy were eligible to participate in the study. Of this eligible population, we randomly re-called a sample of 253 mothers who were recruited to the study after obtaining informed written consent. Females who were currently pregnant or less than six months postpartum and those who have chronic medical problems or acute infective or inflammatory conditions were excluded.

### Data collection procedure

**Fig 1:**
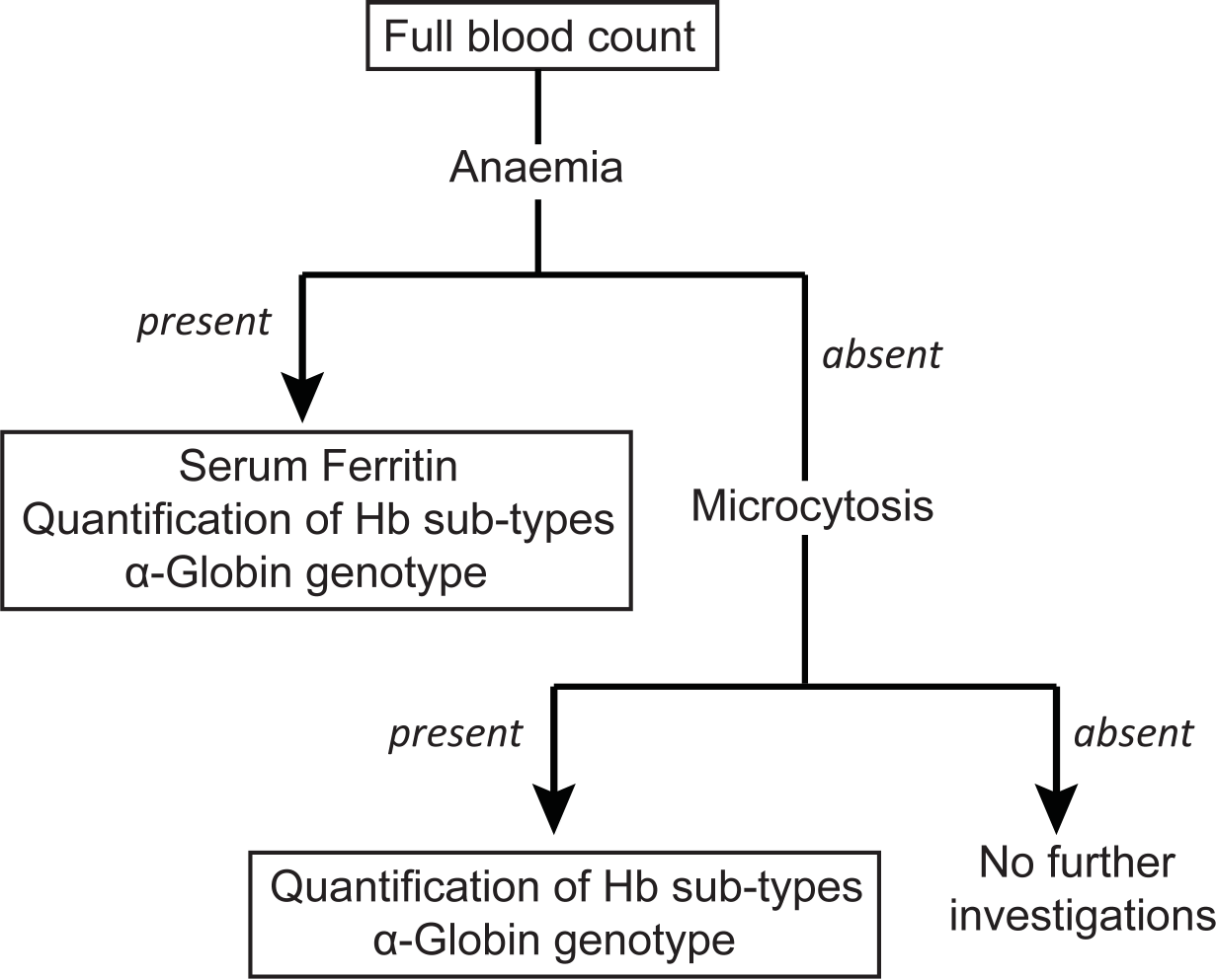
Flow diagram showing investigation protocol.

Data on socio-demographic background, family history, past medical history and present medical problems were collected using an interviewer-administered questionnaire by interviewing participants. Information on perinatal outcome (birth weight and period of gestation at delivery) in relation to the most recent pregnancy was gathered using North Colombo Obstetric Database and were verified during the interview. Then 7.5 ml of venous blood was taken for laboratory investigations of which 2.5 ml was transferred into a plain tube and the remaining sample to two tubes containing EDTA anticoagulant. The EDTA samples were used to perform full blood count, quantification of haemoglobin sub-types and variants and DNA extraction to detect α-globin genotype. Blood collected into plain bottles was used to measure serum ferritin. Full blood counts were performed on all individuals and subsequent investigations were done as shown in Fig 1. Anaemia in women of child bearing age was defined as haemoglobin level <12.0 g/dl and microcytosis was defined as mean corpuscular volume (MCV) <80fL.

### Laboratory procedures

Full blood count and serum ferritin assays were performed in a clinically accredited laboratory (Hamas Hospital Laboratory Services, Ragama, Sri Lanka) using an electrical impedance method (Coulter counter) and chemiluminescent Immunoassay with an analytical sensitivity <0.25 ng/dl respectively. Quantification of haemoglobin sub-types and variants was performed using capillary electrophoresis technique using Capillarys 2 Flex Piercing (Sebia) instrument according to the manufacturer’s instructions. DNA was extracted from the EDTA cell pellet using QIAGEN DNA mini kit and we screened for seven common deletional mutations (α-^3.7^, α-^4.2^, - -^MED^,—-^SEA^,—-^THAI^,—-^FIL^ and—-^20.5^) of the α-globin cluster using a previously described multiplexed polymerase chain reaction [6, 10].

### Statistical analysis

Categorical variables were expressed as counts and percentages and compared using Chi-square test or Fisher’s Exact test. Continuous variables were expressed as mean with standard deviation or median with inter-quartile range. Means were compared using independent sample Student’s t-test and p<0.05 is considered as statistically significant. When analysing the perinatal outcome of thalassaemia trait, individuals who were currently not anaemic were considered as controls and only singleton pregnancies were included. Data were analysed using IBM SPSS 22.0.

### Ethical approval

All participants were adults and informed written consent was obtained before recruiting in to the study. Ethical approval was obtained from Ethics Committee of the University of Kelaniya (P/71/01/2017).

## Results

A total of 3636 deliveries were performed during a period of 21 months between January 2015 and September 2016. Among these, 1396 (38.4%) females were anaemic. Out of the 1396 females who were anaemic during their pregnancy, 253 were chosen at random and re-called for the current study. Eight of them were pregnant again hence, were excluded. Data obtained from the remaining 245 females were analysed. Out of them, 117 (47.8%) remained anaemic (Table 1). Additionally, another 22 (9.0%) females who were not anaemic had microcytosis with low MCV.

**Table 1:** Degree of anaemia and microcytosis in study population.

Parameter	Number (%) (n = 245)
Anaemia (haemoglobin <12.0g/dl)	117 (47.8%)
Mild anaemia (haemoglobin 11.0–11.9g/dl)	80 (32.7%)
Moderate anaemia (haemoglobin 8.0–10.9g/dl)	36 (14.7%)
Severe anaemia (haemoglobin <8.0g/dl)	1 (0.4%)
Microcytosis (Low MCV <80fL)	77 (31.4%)
Microcytic anaemia	55 (22.4%)
Microcytosis without anaemia	22 (9.0%)

### Body iron status

Among the anaemic sub-population, we measured body iron status using serum ferritin. This showed that only 28 (24.8%) females with anaemia were iron deficient. In another 40 (35.4%) females, serum ferritin levels were low-normal (15.1–30.0 ng/ml) suggesting that iron deficiency may be a probable aetiology for anaemia in them without meeting full criteria to diagnose it. Interestingly, low or low-normal serum ferritin levels were observed in a large proportion of females who had normal MCV values (Table 2).

**Table 2:** Body iron status among females with anaemia.

Serum Ferritin Level	Anaemia with low MCV (n = 53)	Anaemia with normal MCV (n = 60)	All anaemia (n = 113)^a^
Low (= <15ng/ml)	15 (28.3%)	13 (21.7%)	28 (24.8%)
Low-normal (15.1–30.0ng/ml)	15 (28.3%)	25 (41.7%)	40 (35.4%)
Normal (30.1–100.0ng/ml)	18 (34.0%)	21 (35.0%)	39 (34.5%)
High (>100.0ng/ml)	5 (9.4%)	1 (1.7%)	6 (5.3%)

^a^ Samples of four subjects (two each with low and normal MCV) were insufficient

### Haemoglobinopathies and α- and β-globin genotypes

All females who had anaemia or low MCV were screened for presence of haemoglobinopathies. Among anaemic females, 18 (15.3%) had some form of β-haemoglobinopathy trait (Table 3); the majority had β-thalassaemia trait, next most common was haemoglobin E trait. None had sickle cell disease or trait. All females who had β-haemoglobinopathy demonstrated low MCV. Screening for α-thalassaemia revealed that it is slightly commoner than β-thalassaemia trait among anaemic females; 20 (17.0%) of the anaemic females had α-thalassaemia trait. Importantly, 6 females with α-thalassaemia trait had normal MCVs. Additionally, 14 (66.0%) females who had low MCVs without anaemia had α-thalassaemia trait. Amongst all females with α-thalassaemia trait, heterozygous α+-thalassaemia due to a 3.7kb deletion (α-^3.7^/αα) was commoner (31/34) than the 4.2kb deletion (α-^4.2^/αα) (2/34). Homozygous α+-thalassaemia trait (α-^3.7^/α-^3.7^) was seen in a single patient and none had α^0^-thalassaemia.

**Table 3:** Frequency of haemoglobinopathy among women with anaemia and/or low MCV.

	Low MCV without anaemia (n = 21)^a^	Anaemia with low MCV (n = 55)	Anaemia with normal MCV (n = 62)	All anaemia (n = 117)
***β-globin genotype***				
Normal	20 (95.2%)	37 (67.3%)	62 (100.0%)	99 (84.7%)
β-thalassaemia trait	1 (4.8%)	15 (27.3%)	0	15 (12.8%)
Haemoglobin E trait	0	2 (3.6%)	0	2 (1.7%)
δβ-thalassaemia trait (probable)	0	1 (1.8%)	0	1 (0.8%)
***α-globin genotype***				
Normal (αα/αα)	7 (33.3%)	41 (74.5%)	56 (90.3%)	97 (82.9%)
Heterozygous α+-thalassaemia trait (α-^3.7^/αα)	14 (66.6%)	12 (21.8%)	5 (8.0%)	17 (14.5%)
Heterozygous α+-thalassaemia trait (α-^4.2^/αα)	0	1 (1.8%)	1 (1.6%)	2 (1.7%)
Homozygous α+-thalassaemia trait (α-^3.7^/α-^3.7^)	0	1 (1.8%)	0	1 (0.8%)

^a^ Sample of one subjects was insufficient

### Interactions between iron status and haemoglobinopathies

**Fig 2:**
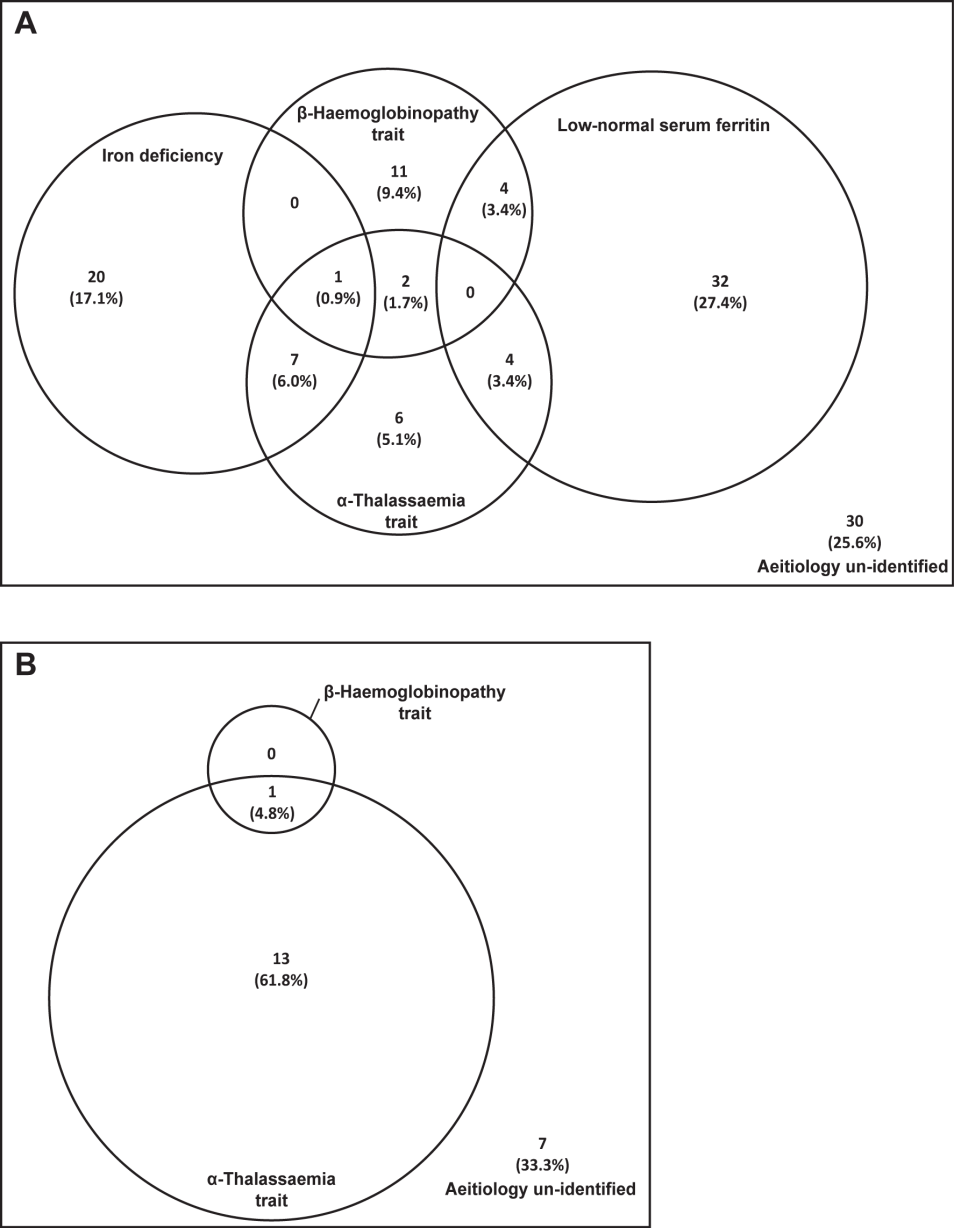
**Interactions between iron deficiency and haemoglobinopathies among females with (A) anaemia and (B) low MCV without anaemia**. Area of each circle represent the relative contribution of each aetiology for anaemia and low MCV without anaemia.

As demonstrated in Fig 2, a number of interactions between aetiologies of anaemia were uncovered in this population. The co-existence of α- or β-thalassaemia with iron deficiency or low-normal serum ferritin were observed in several patients. Furthermore, three individuals in the anaemia group had both α- and β-thalassaemia trait (Fig 2A). Additionally, another patient, who had a low MCV without anaemia, co-inherited α- and β-thalassaemia trait (Fig 2B). Therefore, in this rather small sample of asymptomatic females we were able to diagnose four previously undiagnosed individuals with co-inherited α- and β-thalassaemia suggesting that this co-existence is a more common phenomenon than previously thought. Despite extensive investigations, we were unable to identify the aetiology for anaemia in 30 (25.6%) females.

**Fig 3:**
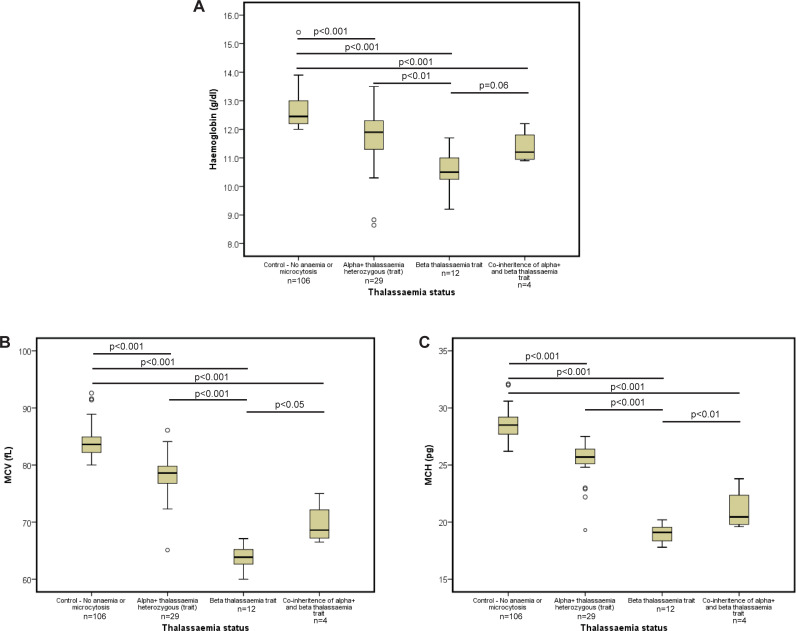
**Distribution of (A) haemoglobin level, (B) MCV and (C) MCH in different heterozygous thalassaemia states.** Each box plot shows interquartile range, middle horizontal bar shows respective median and error bars show range; outliers are marked in circles.

Next we analysed haemoglobin level, MCV and mean corpuscular haemoglobin (MCH) in females with different heterozygous thalassaemia conditions and non-anaemic controls (Fig 3). As previously documented, individuals with β-thalassaemia trait or α-thalassaemia trait showed significantly lower mean haemoglobin, MCV and MCH values compared to controls[11]. Reductions in mean haemoglobin and red blood cell (RBC) indices were more marked in β-thalassaemia trait than in α-thalassaemia trait (p<0.01, p<0.001 and p<0.001 for haemoglobin, MCV and MCH respectively). Individuals who co-inherit both β-thalassaemia trait and α-thalassaemia trait had higher mean haemoglobin values, MCVs and MCHs compared to individuals with only β-thalassaemia (p = 0.06, p<0.05 and p<0.01 for haemoglobin, MCV and MCH respectively). This clearly suggests that clinically significant disease ameliorating interactions are present between α- and β-thalassaemia in the heterozygous states.

### Perinatal outcome of mothers with thalassaemia trait

Finally, we analysed perinatal outcome in relation to the most recent pregnancy of females with thalassaemia trait. This analysis showed that a significantly higher proportion of the off-spring of mothers who have α-thalassaemia trait delivered prematurely (period of gestation <37 weeks) (p<0.01) and had low birth weight (<2500g) (p = 0.054). Similarly, mean birth weight of the off-spring of mothers who have α-thalassaemia trait was significantly lower compared to controls (p<0.05). However, neonatal outcomes of mothers with β-thalassemia trait were comparable to controls (Table 4).

**Table 4:** Birth outcome of off springs of different heterozygous thalassaemia conditions.

Parameter		Significance (compared to controls)
***Birth weight (g)***	***Mean ± SD***	
Control group (n = 102)	3043 ± 479	
β-thalassaemia trait (n = 12)	3210 ± 639	t = 1.06, p = 0.27
α+- thalassaemia trait (α-/αα) (n = 28)	2795 ± 503	t = 2.39, p<0.05
Co-inheritance of α- and β-thalassaemia (n = 3)	3183 ± 350	t = 0.498, p = 0.61
***Low birth weight (<2500g)***	***N*, *prevalence(%)***	
Control group (n = 102)	10 (9.8%)	
β-thalassaemia trait (n = 12)	1 (8.3%)	p = 1.0
α+- thalassaemia trait (α-/αα) (n = 28)	7 (25.0%)	p = 0.054
Co-inheritance of α- and β-thalassaemia (n = 3)	0 (0%)	p = 1.0
***Prematurity (<37 weeks gestation)***	***N*, *prevalence(%)***	
Control group (n = 102)	6 (5.9%)	
β-thalassaemia trait (n = 12)	0 (0%)	p = 1.0
α+- thalassaemia trait (α-/αα) (n = 28)	7 (25.0%)	p<0.01
Co-inheritance of α- and β-thalassaemia (n = 3)	0 (0.0%)	p = 1.0

## Discussion

The main aim of our study was to determine the aetiology of anaemia among females of child bearing age and it was specifically designed to ascertain the contribution of haemoglobinopathies in causing anaemia in this population and thereby in pregnant women. This is particularly important as many health authorities attributes anaemia in pregnancy principally to iron deficiency and advocate universal iron supplementation to pregnant women in resource-poor countries[12]. Our study has shown that these assumptions are incomplete and haemoglobinopathies contribute to up to one-third of anaemia in women of child bearing age at least in this part of the world.

The diagnosis of β-haemoglobinopathy that includes β-thalassaemia, haemoglobin E disease and sickle cell disease, by high performance liquid chromatography or capillary electrophoresis techniques are easy and unequivocal [11]. Facilities for testing this is available free of charge in several centres across Sri Lanka and in fact, pre-marital voluntary testing of all young persons is greatly encouraged through the National Thalassaemia Prevention Programme [13]. Despite this, our study identified eighteen previously un-diagnosed females with β-haemoglobinopathies. This is particularly alarming as all these females carry a risk of giving birth to a child with thalassaemia major if they are married to a partner with β-thalassaemia trait. This emphasises the importance of finding a cause in every pregnant woman who is found to have anaemia during the antenatal period.

In contrast to β-haemoglobinopathies, α-thalassaemia trait cannot be diagnosed in adults without genetic testing and therefore, is greatly under diagnosed in Sri Lanka. However, a recent island-wide survey among adolescents revealed that α-thalassaemia trait is commoner than β-thalassaemia trait in Sri Lanka [7, 10, 14]. Our results collaborate well with the findings of this survey. Another important factor revealed in this study is that a significant proportion of individuals with α-thalassaemia trait had normal haemoglobin levels but were detected because they had low MCVs. In fact, almost two-thirds of females who had non-anaemic microcytosis were found to have α-thalassaemia trait. Similarly, it is known that α-thalassaemia due to single gene deletion can be clinically and haematologically silent and these patients can have normal haemoglobin levels and red cell indices[15]. Therefore, it is possible that we have missed some individuals with α-thalassaemia trait in this population and the prevalence of α-thalassaemia is higher than we report. These findings are particularly important in developing screening programmes for haemoglobinopathy.

Another important finding of this study is the identification of four individuals who co-inherit α- and β-thalassaemia trait. More importantly our results show that the presence of α-thalassaemia trait in individuals with β-thalassaemia trait show favourable effects with comparatively higher haemoglobin, MCV and MCH values. This can be explained by the pathophysiology of thalassaemia which principally relate to the degree of imbalance between α- and β-globin chains in RBCs [16]. The presence of α-thalassaemia trait results in reduced production of α-globin chains thus reducing the α-globin excess which leads to reductions in microcytosis and premature RBC destruction in individuals with β-thalassaemia trait. Our findings confirm the previous reports of ameliorating interactions of α-thalassaemia in patients with β-thalassaemia major and HbE β-thalassaemia [17] as well as in individuals with β-thalassaemia trait [18, 19]. This finding will further strengthen the evidence base supporting a recent attempt to down-regulate α-globin level as a cure for β-thalassaemia [20–22].

We also found a significantly higher prevalence of premature births and low birth weight among offspring of females with α-thalassaemia trait. Although it has been shown previously that anaemia in pregnancy is associated with prematurity and low birth weight [5], reports among patients with thalassaemia trait failed to show an increased risk of both conditions in the offspring [23]. As we have not observed any higher risk of prematurity or low birth weight among β-thalassaemia trait in this study, it may be that this is a phenomenon unique to α-thalassaemia trait. However, we report this observation with caution as it needs confirmation by larger prospective studies in the future.

One potential limitation of this study is that despite extensive investigations we were unable to elucidate the aetiology in approximately one-fourth of individuals with anaemia. Similar findings were reported in the island-wide survey among adolescents[7]. Malaria and helminthiasis have been implicated as causes for anaemia in developing tropical countries however, these infections have extremely low prevalence in Sri Lanka at present [24–26]. In fact, malaria was eliminated from Sri Lanka in 2013 and no cases of indigenous transmission has been reported since then[27]. Therefore, future research should aim at identifying rare causes which might explain anaemia in this group of females.

## Conclusions

In conclusion, we have demonstrated that a large proportion of females with anaemia of child bearing age have haemoglobinopathies and the contribution of α-thalassemia is higher than expected. Both α- and β-thalassaemia trait can co-exist and this co-inheritance has ameliorating effects in the heterozygous states. α-Thalassaemia trait was significantly associated with premature births and low birth weight. These observations should alert policy makers at least in areas where thalassaemia is common to update guidelines and direct physicians to elucidate the causes of anaemia in women of child bearing age and during pregnancy in addition to providing universal iron supplementation.

## Supporting information

S1 File Data set. (XLSX)
